# Synthesis and Antiproliferative Activity of C-3 Functionalized Isobenzofuran-1(*3H*)-ones

**DOI:** 10.3390/molecules18021881

**Published:** 2013-02-01

**Authors:** Róbson Ricardo Teixeira, Gustavo Costa Bressan, Wagner Luiz Pereira, Joana Gasperazzo Ferreira, Fabrício Marques de Oliveira, Deborah Campos Thomaz

**Affiliations:** 1 Departamento de Química, Universidade Federal de Viçosa, Av. P.H. Rolfs, S/N, CEP 36.570-000, Viçosa, MG, Brazil; 2 Departamento de Bioquímica e Biologia Molecular, Universidade Federal de Viçosa, Av. P.H. Rolfs, S/N, CEP 36.570-000, Viçosa, MG, Brazil

**Keywords:** isobenzofuran-1(*3H*)-one, phtalides, antiproliferative activity

## Abstract

A series of thirteen C-3 functionalized isobenzofuran-1(*3H*)-ones (phtalides) was synthesized via condensation, aromatization, and acetylation reactions. NMR (one and two dimensional experiments), IR, and mass spectrometry analysis allowed confirmation of the identity of the synthesized compounds. The substances were submitted to *in vitro* bioassays against U937 (lymphoma) and K562 (myeloid leukemia) cancer cell lines using the MTT cytotoxicity assay. Some derivatives inhibited 90% of cell viability at 100 µM. Also, two phtalides presented biological activity superior than that of etoposide (VP16), a commercial drug used as a positive control in the assays. *In silico* drug properties of the evaluated compounds were calculated and the results are discussed.

## 1. Introduction

**Figure 1:**
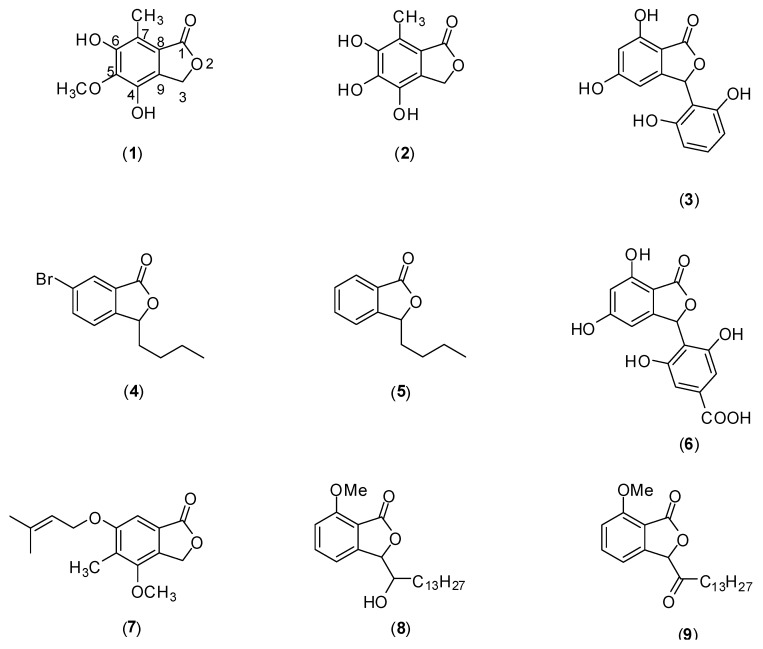
Structures of isobenzofuran-1(*3H*)-ones **1**–**9**.

Several compounds possessing a *γ*-lactone moiety fused to an aromatic ring, known as isobenzofuran-1(*3H*)-ones (phtalides), exhibit many biological activities: antioxidant, presented by compounds **1** and **2** [1]; antifungal, displayed by isopestacin (**3**) [2]; anti-platelet observed with the brominated derivative **4** [3]; and anticonvulsant activity related to compound **5** [4] (Figure 1). The phytotoxic compound **6** [5], the photosynthetic inhibitor **7** [6] and the cytotoxic substances **8** and **9** are other examples of bioactive phtalides. As can be seen in Figure 1, various phtalides are functionalized at C-3 position (see structure **1** for numbering).

The isobenzofuranones **8** and **9** were prepared from anarchadic acids, the major natural phenolic lipid in cashew (*Anacardium occidentale*) nut-shells [7]. Cytotoxicity screening of compounds **8** and **9** against different human cancer cell lines (HL-60 leukemia, SF295 glioblastoma and MDA-MB435 melanoma) by the MTT assay showed that they are active. Compound **8** exhibited low activity against HL-60 (IC_50_ 21.00 µg/mL) and SF295 (IC_50_ > 25 µg/mL) cells, and moderate activity against MDA-MB435 cells (IC_50_ 12.17 µg/mL), while compound **9** exhibited a significant cytotoxic effect against HL-60 cells (IC_50_ 3.24 µg/mL), and moderate activity on SF295 (IC_50_ 10.09 µg/mL) and MDA-MB435 (IC_50_ 8.70 µg/mL). The two compounds exhibited weak to moderate activities against PBMC cells (**8**, IC_50_ > 25 µg/mL; **9**, IC_50_ > 14.51 µg/mL).

A natural extension of this investigation would be the synthesis and cytotoxic screening of isobenzofuranones structurally similar to compounds **8** and **9** but containing alicyclic and aromatic functionalities attached to C-3 position. Considering the aforementioned cytotoxic effects of **8** and **9** and our interest in the development of bioactive compounds [8,9,10,11], we detail in this paper the synthesis and evaluation of the antiproliferative activity of a series of alicyclic and aromatic C-3 functionalized isobenzofuranones. *In silico* drug properties of the evaluated compounds were determined and the results are also discussed.

## 2. Results and Discussion

### 2.1. Preparation of Isobenzofuran-1(3H)-ones

**Scheme 1:**
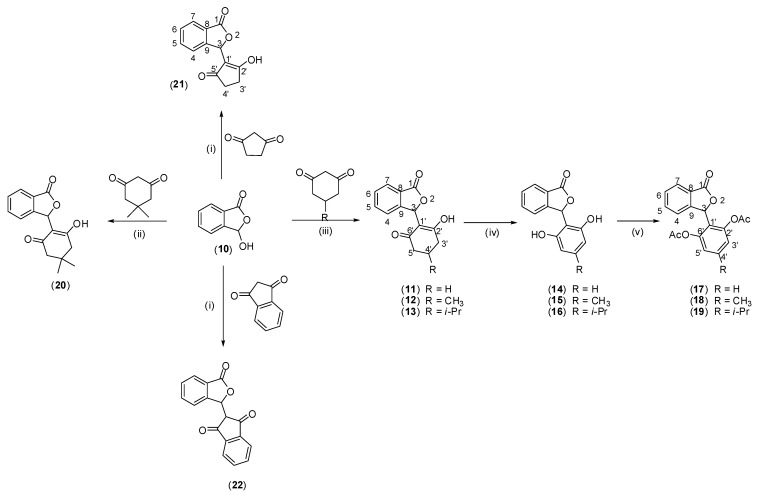
Synthetic route involved in the preparation of compounds **11**–**22**.

The synthetic route used to prepare compounds **11**–**22**, functionalized at C-3 position of the isobenzofuranone core, is outlined in Scheme 1.

**Scheme 2:**
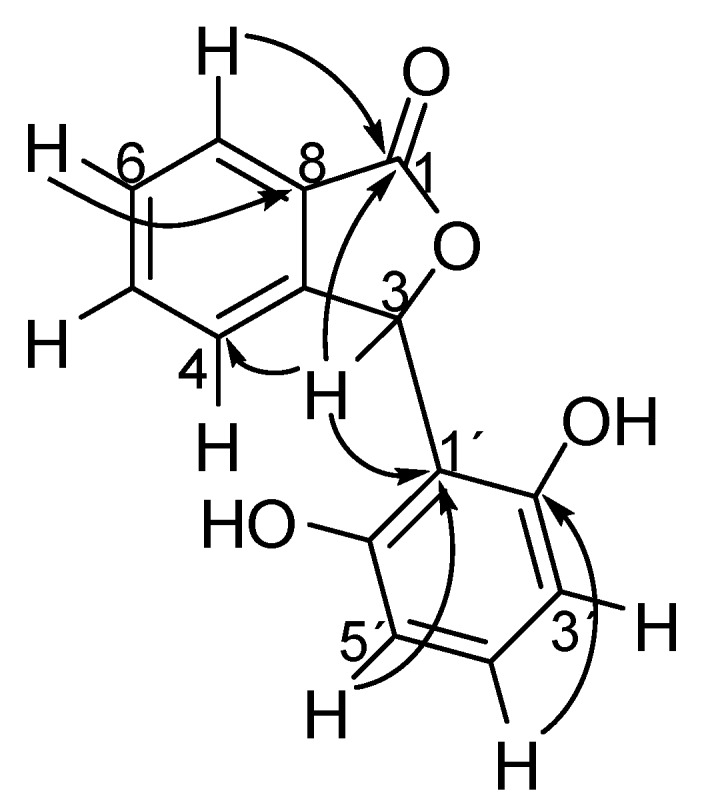
Some long range correlations found in the HMBC contour plot of compound **14**.

DBU-promoted condensation of commercially available phthalaldehydic acid (**10**) and different 1,3-dicarbonyl compounds afforded derivatives **11**–**13** and **20**–**22** in yields varying from 53 to 95% [12,13]. Phenolic compounds **14**–**16** were prepared via Hg(OAc)_2_ mediated aromatization [14]. Treatment of substances **14**–**16** with Ac_2_O/DMAP resulted in the formation of acetylated derivatives **17**–**19**. The compounds were fully characterized by IR, NMR, and MS analysis. High resolution mass spectrometry confirmed the molecular formula of the compounds. A combination of two dimensional NMR analyses (HSQC and HMBC) allowed complete hydrogen and carbon assignments. Taking compound **14** into consideration, some of the major long-range correlations (*J^2^* and *J*^3^) observed in the HMBC contour plot are depicted in Scheme 2.

### 2.2. Evaluation of Antiproliferative Activity

**Figure 2:**
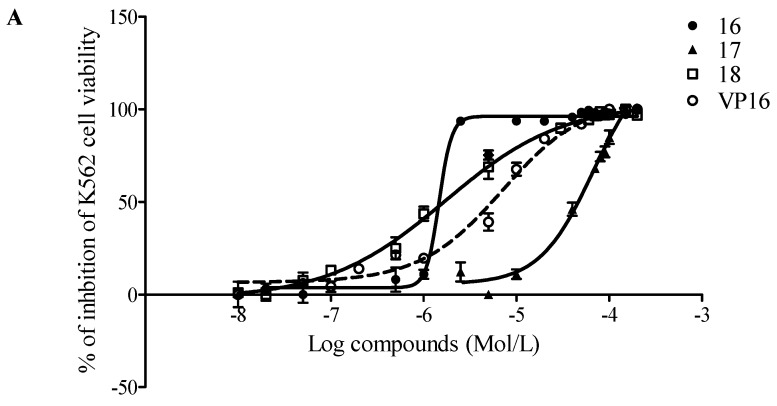

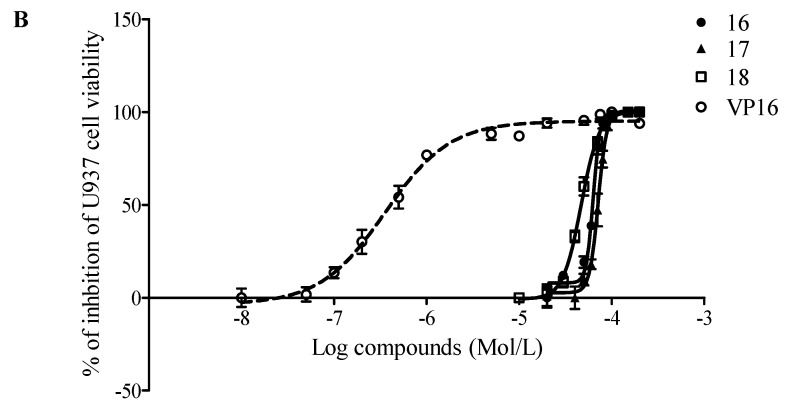
Dose-response profiles of compounds **16**–**18** and positive control VP16 on viability of K562 (**A**) and U937 (**B**) cells. K562 and U937 cell lines were treated for 48 h with increasing amounts of the indicated substances. All the assays were performed in quadruplicates and the results were normalized considering the cultures treated with the vehicle alone (1% DMSO).

Isobenzofuran-1(*3H*)-ones **10**–**22** were biologically evaluated against U937 (lymphoma) and K562 (myeloid leukemia) cell lines in comparison to DMSO (1%) and etoposide (VP16), used as negative and positive controls, respectively. After 48 h of treatment, compounds **16**–**18** were the most effective in decreasing the viability of both the cell lines tested (Table 1 and Table 2). Compound **19** also inhibited proliferation, though only against K562 cells. Although the IC_50_ values obtained for compounds **16**–**18** suggest a moderate effect on U937 cells, a strong inhibitory activity was observed for compounds **16** and **18** against K562 cells (IC_50_ 2.79 and 1.71 µM, respectively; Table 3 and Figure 2). More importantly, these inhibitory activities were even higher than those observed for etoposide (VP16) (IC_50_ 7.06 µM for K562), an anticancer drug used in several chemotherapy regimes, including against leukemia.

### 2.3. Structure-Activity Relationship and in Silico Calculations of Drug-Like Properties

Considering the therapeutic potential of the isobenzofuranone derivatives described so far, a computational study was carried out to predict the physical chemical properties of compounds **10**–**22**. These parameters influence pharmacokinetic properties, such as absorption, distribution, metabolism, and excretion (ADME). A screening was conducted to evaluate whether the substances can fulfill the characteristics of candidate drugs, based on Lipinski’s Rule of Five [15] and other related criteria added later by Veber and co-workers [16]. These characteristics theoretically determine if a compound presents absorptivity on and permeability across membranes, properties that would likely make it an orally active drug in humans.

Molecular attributes analyzed were *n*-octanol/water partition coefficient (LogP ≤ 5), the amount of hydrogen bond donors (HBD ≤ 5), the amount of hydrogen bond acceptor (HBA ≤ 10), the molecular weight of the drugs (MW ≤ 500), number of rotatable bonds (nRotb < 10) and polar surface area (PSA < 140 Å^2^). Values in parentheses represent the ideal values according to Lipinski [15] and Veber [16]. Physicochemical parameters were calculated by using Osiris Property Explorer [17] and Molinspiration [18], good free computational tools which help to predict pharmacokinetic properties of candidate drugs used by several research groups [19,20,21,22].

The Osiris Property Explorer allows evaluation of chemical and physicochemical data that influence the pharmacokinetic properties of a drug. The Osiris calculations for toxicity and drug-likeness follow a fragment-based approach. The software can calculate lipophilicity, inferred from LogP value, solubility in water, expressed as LogS, molecular weight, drug-likeness indices and drug scores. Low hydrophilicity, and therefore high LogP values, may cause poor absorption or permeation. Also, Osiris software calculates various attributes of the drugs, such as toxicity risks (mutagenic, tumorigenic, irritant and reproductive effects), drug likeness, and drug score [23,24]. Calculation of LogP and molecular weight, as well as total polar surface area (TPSA), number of rotatable bonds (nRotB), number of hydrogen bond donor (HBD), number of hydrogen bond acceptor (HBA) and bioactivity scores [25] can also be determined in the Molinspiration package. Table 4 and Table 5 show the results of the calculations based on these computational packages.

A parameter to determine good absorption of compounds is the value of cLogP. On this basis, for a reasonable probability of good absorption, LogP value must not be greater than 5.0. Notably, the cLogP values of phtalides **10**–**22** are greater than the etoposide value, suggesting that the hydrophobicity of isobenzofuranone derivatives is greater than that of the compound used as positive control in the biological assays. Normally, the drugs interacting with enzyme inside the human body have LogP values between 2 and 5 [26]. In this respect, most of the studied compounds present values within this range (Table 4 and Table 5).

Toxicity risk alerts are an indication that the structure of a compound may be harmful. Analysis of theoretical toxicity risks for these series using Osiris software revealed that all the compounds examined were non-mutagenic, non-tumorigenic, non-irritating and without effects on mammalian reproduction (Table 4). In addition, these compounds were evaluated as potential drugs through the drug-likeness calculations, showing negative values (between −8.59 and −3.58, Table 4), indicating that compounds **10**–**22** do not contain fragments that are frequently present in commercial drugs [23,24].

Aqueous solubility of a compound significantly affects its absorption and distribution characteristics. In general, it is desirable to obtain compounds which are soluble in a biological environment. A parameter to evaluate the solubility is LogS. More than 80% of the drugs in the market have estimated LogS values greater than −4 [19]. In the case of compounds **19** and **22**, LogS values (−4.41 and −4.04, respectively) are lower, compared to others in the series (Table 4).

Drug score values can be used to judge the compound’s overall potential to qualify it for a drug. The values are the combination of drug likeness, toxicity risk, and some physicochemical parameters, such as cLogP, solubility, and molecular weight [23,24]. These values would be used to evaluate the potential of a candidate drug. As can be seen in Table 4, drug score values of compounds **10**–**22** are comparable or higher in relation to the commercial drug etoposide.

Hydrogen-bonding capacity (the number of HBA) was estimated by considering the number of nitrogen and oxygen atoms in the chemical structure. The number of HBD atoms was the sum of hydrogen atoms bound to oxygen and to nitrogen atoms [23]. The values of these parameters for compounds **10**–**22** are within the limit set by the Lipinski rules. This rule also suggests that two or more violations in a compound show probability of bio-availability problems [27]. All the compounds in Table 4 present zero violations of the rules, unlike epotoside, that has two rule violations.

In addition to LogP, total polar surface area (TPSA) is an important descriptor for prediction of drug transport properties, including intestinal absorption, Caco-2 monolayer permeability, and blood-brain barrier penetration [28,29]. This parameter was calculated using Molinspiration software based on the sum of the surface belonging to polar atoms (usually oxygens, nitrogens, and attached hydrogens) [25]. Compounds with TPSA ≥ 140 A^2^ are thought to have poor oral bioavailability and TPSA ≤ 61 A^2^ are thought to have good bioavailability [28]. Considering the TPSA values, compounds **10**–**22** (Table 5) are expected to exhibit good bioavailability, based on the acceptable range. However, it must be mentioned that TPSA ≤ 140 A^2^ is a necessary but not sufficient criterion for good bioavailability.

The number of rotatable bonds is a simple topological parameter of molecular flexibility. It has been shown to be a very good descriptor of oral bioavailability of drugs [17]. Compounds **17**, **18** and **19** exhibit greater flexibility among the compounds tested with nRotB = 3, 3, and 4, respectively, while the commercial drug etoposide presented a value equal to 5 (Table 5).

Bioactivity of all the 13 compounds and etoposide were analyzed under different regular human body receptors, with six criteria of known successful drug activity being observed in the areas of GPCR ligand activity, ion channel modulation, kinase inhibition activity, nuclear receptor ligand activity, protease inhibition activity, and enzyme inhibition activity. The higher the value of the score, the better the probability of a molecule being active. Results of these parameters are presented (Table 5) by numerical assignments. Like etoposide, all the compounds studied have consistent negative numerical values for ion channel modulator and kinase inhibitor. In the other categories, the values for some isobenzofuranones were positive.

Although no plane correlation could be found between the physical chemical properties of isobenzofuranone derivatives and antiproliferative activity, some generalizations can be made. For instance, the presence of the acetyl group on the benzene ring of compounds **14**–**19** seems to increase biological activity. There is an apparent correlation between the values of cLogP with the antiproliferative activity of these compounds, since the most active compounds have cLogP values in the range of 3.1–4.19. The other compounds show lower lipophilicity values.

## 3. Experimental

### 3.1. General

Analytical grade phthalaldehydic acid, 8-diazabicyclo [5.4.0]undec-7-ene (DBU), cycloexane-1,3-dione, 5-methylcyclohexane-1,3-dione, 5,5-dimethyl cicloexane-1,3-dione (dimedone), 5-isopropylcyclohexane-1,3-dione and dymethylaminopyridine (DMAP) were purchased from Aldrich (St. Louis, MO, USA). Mercury (II) acetate, sodium acetate, acetic acid, acetonitrile and chloroform were purchased from Vetec (Rio de Janeiro, Brazil). ^1^H and ^13^C-NMR spectra were recorded on a Bruker AVANCE DRX 400 spectrometer at 400 and 100 MHz, respectively, using MeOH-*d_4_*, CDCl_3_ and DMSO-*d_6_* as solvents. Infrared spectra were recorded on a Varian 660-IR, equipped with GladiATR scanning from 4000 to 500 cm^−1^. HRMS data were recorded under ESI conditions on a micrOTOF-QII Brucker spectrometer. Melting points are uncorrected and were obtained with a MQAPF-301 melting point apparatus (Microquimica, Campinas, Brazil). Analytical thin layer chromatography was carried out on TLC plates recovered with 60GF254 silica gel. Column chromatography was performed over silica gel (60–230 mesh).

### 3.2. Synthesis of Compounds ***11**–**13***, Exemplified by the Synthesis of 3-(2-Hydroxy-6-oxocyclohex-1-enyl)isobenzofuran-1(3H)-one *(**11**)*

Cyclohexane-1,3-dione (0.891 g, 7.95 mmol), acetonitrile (10 mL) and DBU (1.06 mL, 7.1 mmol) were added to a round-bottomed flask (50 mL). The mixture was magnetically stirred at room temperature for 5 min. Phthaldehydic acid (**10**, 1.070 g; 7.1 mmol) was added and the mixture was refluxed for 7 h. The reaction was quenched by the addition of aqueous 10% HCl (10 mL), followed by ethyl acetate (100 mL). The layers were separated and the organic layer was washed with water (2 × 20 mL) and brine (10 mL), dried over anhydrous sodium sulphate and concentrated under reduced pressure. The resulting material was recrystallized from ethyl acetate. Compound **11** was obtained as a white solid in 64% yield (1.121 g; 4.6 mmol). The structure of the compound is supported by the following data: TLC R_f_ = 0.20 (ethyl acetate). mp 217.5–218.0 °C. IR (ATR, cm^−1^): 2960, 2917, 2887, 2555 (broad band), 1758, 1565, 1380, 1281, 1056, 1025, 944. ^1^H-NMR (400 MHz, DMSO-*d_6_*): *δ* 1.83–1.91 (m, 2H, H-4′), 2.26–2.45 (m, 4H, H-3′/H-5′), 6.59 (s, 1H, H-3), 7.30 (d, 1H, *J* = 7.6 Hz, H-4), 7.50 (dd, 1H, *J* = 7.6, 7.2 Hz, H-5), 7.65 (dd, 1H, *J* = 7.6, 7.2 Hz, H-6), 7.78 (d, 1H, *J* = 7.6 Hz, H-7). ^13^C-NMR (100 MHz, DMSO-*d_6_*): *δ* 20.1 (C-3′/C-5′), 32.6 (C-4′), 74.3 (C-3), 109.1 (C-1′), 121.4 (C-4), 124.2 (C-7), 126.5 (C-8), 128.0 (C-6), 133.6 (C-5), 150.7 (C-9), 170.7 (C-1), 188.0 (C-2′). HREIMS *m/*z (M+H^+^): Calculated for C_14_H_13_O_4_, 245.0814; found: 245.0840.

Compounds **12** and **13** were prepared using a procedure similar to that described for compound **11**.

*3-(2-Hydroxy-4-methyl-6-oxocyclohex-1-enyl)isobenzofuran-1(3H)-one* (**12**): The compound was obtained as a white solid in 68% yield. The structure of the compound is supported by the following data: TLC R_f_ = 0.50 (ethyl acetate). mp. 214.6–218.3 °C. IR (ATR, cm^−1^): 2960, 2917, 2886, 2565 (broad band), 1757, 1564, 1380, 1281, 1060, 944, 787, 740, 690, 538. ^1^H-NMR (400 MHz, MeOH-*d_4_*): *δ* 1.08 (d, 3H, *J* = 4.8, -CH_3_), 2.13–2.55 (m, 5H, H-4′, H-3′/H-5′), 6.69 (s, 2H, H-3), 7.31 (d, 1H, *J* = 7.6 Hz, H-4), 7.49 (dd, 1H, *J* = 7.6, 7.2 Hz, H-5), 7.64 (dd, 1H, *J* = 7.6, 7.2 Hz, H-6), 7.81 (d, 1H, *J* = 7.6 Hz, H-7); ^13^C-NMR (100 MHz, MeOH-*d_4_*): *δ* 21.0 (-CH_3_), 29.5 (C-3′/C-5′), 42.2 (C-4′), 76.6 (C-3), 110.8 ( C-1′), 122.7 (C-4), 125.7 (C-7), 128.2 (C-8), 129.5 (C-6), 135.2 (C-5), 152.4 (C-9), 174.2 (C-1), 189.7 (C-2′). HREIMS *m/*z (M+H^+^): Calculated for C_15_H_15_O_4_, 259.0970; found: 259.0998.

*3-(2-Hydroxy-4-isopropyl-6-oxocyclohex-1-enyl)isobenzofuran-1(3H)-one* (**13**): The compound was obtained as a white solid in 69% yield. The structure of the compound is supported by the following data: TLC R_f_ = 0.08 (hexane-ethyl acetate 1:3 v/v). mp 176.7–177.8 °C. IR (ATR, cm^−1^): 2964, 2872, 2534 (broad band), 2034, 1758, 1555, 1466, 1370, 1341, 1252, 1090, 1063, 950, 727. ^1^H-NMR (300 MHz, MeOH-*d_4_*): *δ* 0.94 (d, 6H, *J* = 6.9 Hz, -CH(CH_3_)_2_), 1.57–1.67 (m, 1H, -CH(CH_3_)_2_), 1.83–1.96 (m, 1H, H-4′), 2.22–2.51 (m, 4H, H-3′ and H-5′), 6.68 (s, 1H, H-3), 7.31 (dd, 1H, *J* = 7.5, 0.9, H-4), 7,50 (t, 1H, *J* = 7.5, H-5), 7.64 (td, 1H, *J* = 7.5, 1.2, H-6), 7.80 (d, 1H, *J* = 7.5, H-7). ^13^C-NMR (75 MHz, MeOH-*d_4_*): *δ* 18.7 (-CH(CH_3_)_2_), 31.7 (-CH(CH_3_)_2_), 36.8 (C-3′/C-5′), 39.5 (C-4′), 75.3 (C-3), 109.4 (C-1′), 121.4 (C-4), 124.4 (C-7), 126.8 (C-8), 128.2 (C-6), 139.9 (C-5), 151.0 (C-9), 172.9 (C-1), 189,4 (C-2′). HREIMS *m/*z (M+H^+^): Calculated for C_17_H_19_O_4_, 287.1283; found: 287.1260.

### 3.3. Synthesis of Compounds ***14**–**16***, Exemplified by the Synthesis of 3-(2,6-Dihydroxyphenyl)-isobenzofuran-1(3H) one *(**14**)*

A 50 mL round-bottomed flask was charged with compound **11** (477 mg, 1.95 mmol), acetic acid (20 mL), Hg(OAc)_2_ (1,897 mg; 5.93 mmol) and NaOAc (481 mg, 5.86 mmol). The resulting mixture was stirred under reflux for 5 h. The reaction mixture was cooled and 5 mL of HCl 1 mol L^−1^ was added and stirred for an additional 15 min. The mixture was filtered through a Celite pad and the filtrate diluted with ethyl acetate (60 mL). The organic layer was separated, washed with saturated sodium bicarbonate aqueous solution (2 × 10 mL), water (5 mL), brine (5 mL) and 0.200 mol L^−1^ sodium EDTA aqueous solution (2 × 15 mL). The organic layer was dried over sodium sulphate, filtered, concentrated under reduced pressure and the residue purified by silica gel column chromatography (hexane-ethyl acetate; 1:3 v/v). Compound **11** was obtained as a white solid in 68% yield (320 mg; 1.32 mmol). The structure of the compound is supported by the following data: TLC R_f_ = 0.70 (hexane-ethyl acetate 1:3 v/v). mp 238.6–239.8 °C. IR (ATR, cm^−1^): 3470 (broad band), 3296 (broad band), 1712, 1614, 1469, 1011, 945, 715, 587. ^1^H-NMR (400 MHz, CDCl_3_ + DMSO-*d_6_*): *δ* 6.29 (d, 2H, *J* = 8.0 Hz, H-3′/H-5′), 6.92 (t, 1H, *J* = 8.0 Hz, H-4), 7.02 (s, 1H, H-3), 7.31 (d, 1H, *J* = 7.2 Hz, H-4), 7.47 (t, 1H, *J* = 7.6 Hz, H-6), 7.59 (t, 1H, *J* = 7.2 Hz, H-5), 7.81 (d, 1H, *J* = 7.6 Hz, H-7). ^13^C-NMR (100 MHz, CDCl_3_ + DMSO-*d_6_*): *δ* 75.6 (C-3), 106.9 (C-3′/C-5′), 108.6 (C-1′), 121.8 (C-4), 124.2 (C-7), 127.2 (C-8), 127.9 (C-6), 130.2 (C-4′), 133.3 (C-5), 151.1 (C-9), 157.5 (C-2′/C-6′), 171.5 (C-1). HREIMS *m/*z (M+H^+^): Calculated for C_14_H_11_O_4_, 243.0657; found: 243.0658.

Compounds **15** and **16** were prepared using a procedure similar to that described for compound **14**.

*3-(2,6-Dihydroxy-4-methylphenyl)-isobenzofuran-1(3H)-one* (**15**): The compound was obtained as a pale yellow solid via aromatization of compound **12** in 71% yield (purified by silica gel column chromatography, using hexane-ethyl acetate 1:3 v/v as the eluting solvent). The structure of the compound is supported by the following data: TLC R_f_ = 0.82 (hexane-ethyl acetate 1:3 v/v). mp 228–231 °C. IR (ATR, cm^−1^): 3579, 3201 (broad band), 2924, 2853, 1721, 1618, 1595, 1305, 1046, 952, 821, 734, 684, 535. ^1^H RMN (200 MHz, CDCl_3_ + MeOH-*d_4_*): *δ* 1.94 (s, 3H, -CH_3_), 5.94 (s, 2H, H-3′/H-5′), 6.82 (s, 1H, H-3), 7.11 (d, 1H, *J* = 7.2 Hz, H-4); 7.16–7.44 (m, 2H, H-5 e H-6), 7.62 (d, 1H, *J* = 7.2 Hz, H-7). ^13^C-NMR (50 MHz, CDCl_3_ + MeOH-*d_4_*): *δ* 22.5 (-CH_3_), 72.2 (C-3), 106.9 (C-1′), 109.1 (C-3′/C-5′), 123.2 (C-4), 125.4 (C-6), 128.5 (C-8), 128.9 (C-7), 134.4 (C-5), 141.9 (C-4′), 152.6 (C-9), 158.3 (C-2′/C-6′), 173.2 (C-1). HREIMS *m/*z (M+H^+^): Calculated for C_15_H_13_O_4_, 257.0814; found: 257.0880.

*3-(2,6-Dihydroxy-4-isopropylphenyl)-isobenzofuran-1(3H)-one* (**16**): The compound was obtained as a viscous orange oil via aromatization of compound **13** in 94% yield (purified by silica gel column chromatography, using hexane-ethyl acetate 1:1 v/v as the eluting solvent). The structure of the compound is supported by the following data: TLC R_f_ = 0.44 (hexane-ethyl acetate 1:1 v/v). IR (ATR, cm^−1^): 3305 (broad band), 2959, 2924, 2870, 2159, 1726, 1621, 1596, 1433, 1034, 732. ^1^H-NMR (300 MHz, MeOH-*d_4_*): *δ* 1,17 (d, 6H, *J* = 6.9 Hz, -CH(CH_3_)_2_), 2.68 (hept, 1H, *J* = 6.9 Hz; -CH(CH_3_)_2_), 6.17 (s, 2H; H-3′/H-5′), 7.02 (s, 1H, H-3); 7.30 (dd, 1H, *J* = 7.5, *J* = 0.9 Hz, H-4), 7.49 (t, 1H, *J* = 7.2 Hz, H-5), 7.63 (td, 1H, *J* = 7.5, 1.2 Hz, H-6), 7.82 (d, 1H, *J* = 7.5 Hz, H-7). ^13^C-NMR (75 MHz, MeOH-d_4_) *δ*: 22.9 (-CH(CH_3_)_2_), 34.2 (-CH(CH_3_)_2_), 76.8 (C-3), 104.6 (C-3/C-5′), 105.8 (C-1′), 121.8 (C-4), 124.1 (C-6), 127.0 (C-8), 128.0 (C-7), 133.7 (C-5), 151.8 (C-9), 152.3 (C-4′), 157.6 (C-2′/C-6′), 173.4 (C-1). HREIMS *m/*z (M+H^+^): Calculated for C_17_H_17_O_4_, 285.1127; found: 285.1116.

### 3.4. Synthesis of Compounds ***17**–**19***, Exemplified by the Synthesis of 2-(3-Oxo-1,3-dihydroisobenzofuran-1-yl)-1,3-phenylene diacetate *(**17**)*

A round-bottomed flask (25 mL) was charged with compound **14** (0.109 g, 0.45 mmol), chloroform (5 mL), acetic anhydride (0.16 mL, 1.62 mmol), and a catalytic amount of DMAP (0.005 g, 0.045 mmol). The reaction mixture was stirred at room temperature for 12 h. The solvent was removed and compound **17** was purified by recrystallization from ethyl acetate affording a white solid in 44% yield (0.064 g, 0.20 mmol). The structure of the compound is supported by the following data: TLC R_f_ = 0.54 (hexane-ethyl acetate 1:1 v/v). mp. 175.9–177.8 °C. IR (ATR, cm^−1^): 1756, 1611, 1465, 1369, 1285, 1170, 1023, 975; ^1^H-NMR (200 MHz, DMSO-*d_6_*): *δ* 2.01 (brs, 6H, 2 × -COCH_3_), 6.85 (s, 1H, H-3), 7.08 (d, 2H, *J* = 8.2 Hz, H-3′/H-5′), 7.25 (d, 1H, *J* = 6.8 Hz, H-4), 7.53 (t, 1H, *J* = 8.2 Hz, H-4′), 7.58–7.75 (m, 2H, H-5 and H-6), 7.95 (d, 1H, *J* = 7.4 Hz, H-7). ^13^C-NMR (50 MHz, DMSO): *δ* 22.0 (-COCH_3_), 76.0 (C-3), 122.3 (C-3′/C-5′), 123.2 (C-1′), 124.4 (C-4), 126.6 (C-7), 127.3 (C-8), 131.2 (C-6), 132.5 (C-4′), 136.5 (C-5), 150.67 (C-9), 151.61 (C-2′/C-6′), 170.5 (C-1), 172.0 (-COCH_3_). HREIMS *m/*z (M+H^+^): Calculated for C_18_H_15_O_6_, 327.0869; found: 327.0848.

Compounds **18** and **19** were prepared using a procedure similar to that described for compound **17**.

*5-Methyl-2-(3-oxo-1,3-dihydroisobenzofuran-1-yl)-1,3-phenylene diacetate* (**18**): The compound was obtained as a pale yellow solid from compound **15** in 93% yield (after purification by silica gel column chromatography, using hexane-ethyl acetate 1:2 v/v as the eluting solvent). The structure of the compound is supported by the following data: TLC R_f_ = 0.8 (hexane-ethyl acetate 1:2 v/v) mp. 171.5–172.3 °C. IR (ATR, cm^−1^): 2925, 2855, 1758, 1621, 1172, 1040, 974, 692, 738. ^1^H-NMR (200 MHz, DMSO-*d_6_*): *δ* 1.99 (brs, 6H, 2 × -COCH_3_), 2.31 (s, 3H, CH_3_), 6.78 (s, 1H, H-3), 6.94 (s, 2H, H-3′/H-5′), 7.23 (d, 1H, *J* = 7.4 Hz, H-4), 7.61 (dd, 1H, *J* = 7.4, 7.2 Hz, H-5), 7.73 (dd, 1H, *J* = 7.4, 7.2 Hz, H-6), 7.93 (d, 1H, *J* = 7.2 Hz, H-7). ^13^C-NMR (50 MHz, DMSO-*d_6_*): *δ* 22.0 (-COCH_3_), 22.3 (-CH_3_), 76.0 (C-3), 119.3 (C-3′/C-5′), 123.6 (C-1′), 124.4 (C-4), 126.5 (C-6), 127.3 (C-8), 131.0 (C-7), 136.4 (C-5), 142.9 (C-9′), 150.8 (C-4′), 151.4 (C-2′/C-6′), 170.5 (C-1), 171.9 (-COCH_3_). HREIMS *m/*z (M+H^+^): Calculated for C_19_H_17_O_6_, 341.1025; found: 341.1030.

*5-Isopropyl-2-(3-oxo-1,3-dihydroisobenzofuran-1-yl)-1,3-phenylene diacetate* (**19**): This compound was obtained as a solid in 78% yield (after purification by recrystallization from ethyl acetate) from compound **15**. The structure of the compound is supported by the following data: TLC R_f_ = 0.81 (hexane-ethyl acetate 1:1 v/v). mp. 157.5–158.1 °C. IR (ATR, cm^−1^): 2963, 2930, 2870, 1756, 1620, 1366, 1288, 1179, 1030, 973, 739, 562. ^1^H-NMR (200 MHz, DMSO-*d_6_*): *δ* 1.16 (d, 1H, *J* = 7.0 Hz, -CH(CH_3_)_2_), 1.98 (brs, 6H, 2 × -COCH_3_), 2.89 (hept, 1H, *J* = 7.0 Hz, -CH(CH_3_)_2_, 6.78 (S, 1H, H-3), 7.00 (s, 2H, H-3′/H-5′), 7.26 (d, 1H, *J* = 7.2 Hz, H-4), 7.54–7.78 (m, 2H, H-5 and H-6), 7.93 (d, 1H, *J* = 7.2 Hz, H-7). ^13^C-NMR (50 MHz, DMSO-*d_6_*): *δ* 22.0 (-COCH_3_), 25.0 (-CH(CH_3_)_2_), 34.80 (-CH(CH_3_)_2_), 76.0 (C-3), 119.6 (C-3′/C-5′), 121.0 (C-1′), 124.5 (C-4), 126.5 (C-6), 127.3 (C-8), 131.1 (C-7), 136.4 (C-5), 150.7 (C-9), 151.5 (C-2′/C-6′), 153.9 (C-4′) 170.5 (C-1), 171.9 (-COCH_3_). HREIMS *m/*z (M+H^+^): Calculated for C_21_H_21_O_6_, 369.1338; found: 369.1418.

### 3.5. Synthesis of 3-(2-Hydroxy-4,4-dimethyl-6-oxo-cyclohexen-1-yl)isobenzofuran-1(3H)-one *(**20**)*

Dimedone (500 mg; 3.57 mmol), chloroform (5 mL), and DBU (0.53 mL; 3.57 mmol) were placed into a 25 mL round-bottomed flask. The resulting mixture was stirred at room temperature for 5 min followed by the addition of 5-hydroxyisobenzofuran-1(*3H*)-one (**19**) (495 mg; 3.30 mmol). The reaction mixture was stirred at room temperature for 5 h, quenched with 10% HCl (5 mL), and diluted with ethyl acetate (100 mL). The organic layer was separated, washed with water (2 × 20 mL) and brine (10 mL), dried over sodium sulphate, filtered and concentrated under reduced pressure. Compound **18** was obtained as a white solid in 95% yield (850 mg; 3.12 mmol) after recrystallization from ethyl acetate. The structure of the compound is supported by the following data: TLC R_f_ = 0.10 (hexano-ethyl acetate 1:1 v/v). mp 212.1–213.0 °C. IR (ATR, cm^−1^): 2962, 2934, 2884, 2535 (broad band), 1763, 1569, 1382, 1321, 1121, 1059, 942, 738, 694, 613, 574. ^1^H-NMR (400 MHz, MeOH-*d_4_*): *δ* 1.09 (s, 6H, 2 × CH_3_), 2.35 (s, 4H, H-3′/H-5′), 6.70 (s, 1H, H-3), 7.31 (d, 1H, *J* = 7.6 Hz, H-4), 7.50 (t, 1H, *J* = 7.6 Hz, H-5), 7.66 (t, 1H, *J* = 7.6 Hz, H-6), 7.81 (d, 1H, *J* = 7.6 Hz, H-7); ^13^C-NMR (100 MHz, MeOH-*d_4_*): *δ* 28.5 (-CH_3_), 33.1 (C-4′), 47.9 (C-3′/C-5′), 76.6 (C-3), 110.1 (C-1′), 122.6 (C-4), 125.8 (C-7), 128.2 (C-8), 129.5 (C-6), 135.2 (C-5), 152.4 (C-9), 174.2 (C-1), 188.9 (C-2′). HREIMS *m/*z (M+H^+^): Calculated for C_16_H_17_O_4_, 273.1127; found: 273.1124.

### 3.6. Synthesis of Compounds ***21*** and ***22***, Exemplified by the Synthesis 3-(2-Hydroxy-5-oxocyclopent-1-enyl)isobenzofuran-1(3H)-one *(**21**)*

Cyclopentane-1,3-dione (0.357 g; 3.64 mmol), chloroform (5 mL), and DBU (0.56 mL, 3.70 mmol) were added to a 25 mL round-bottomed flask. The mixture was stirred at room temperature for 5 min. Phthalaldehydic acid (**10**) (0.533 g, 3.55 mmol) was added and the resulting mixture was refluxed for 5 h, quenched with 10% HCl (5 mL), and diluted with ethyl acetate (100 mL). The organic layer was separated, washed with water (2 × 20 mL) and brine (10mL), dried over sodium sulphate, filtered and concentrated under reduced pressure. Compound **21** was obtained as a white solid in 53% yield (0.448 g, 1.94 mmol) after recrystallization from ethyl acetate. The structure of the compound is supported by the following data: TLC R_f_ = 0.10 (hexane-ethyl acetate 1:1 v/v). mp. 203.6–205.2 °C. IR (ATR, cm^−1^): 2987, 2930, 2370 (broad band), 2366, 2339, 1763, 1648, 1557, 1368, 1281, 1048, 963. ^1^H-NMR (200 MHz, CDCl_3_ + DMSO-*d_6_*) *δ* 2.27 (brs, 4H, 2 × -CH_2_), 6.07 (s, 1H, H-3), 7.11 (d, 1H, *J* = 7.2 Hz, H-4), 7.23–7.45 (m, 2H, H-5 and H-6), 7.62 (d, 1H, *J* = 7.2 Hz, H-7). ^13^C-NMR (50 MHz, CDCl_3_ + DMSO-*d_6_*): *δ* 31.8 (-CH_2_), 74.5 (C-3), 112.6 (C-1′), 123.3 (C-4), 126.0 (C-7), 127.5 (C-8), 129.7 (C-6), 134.9 (C-5), 150.1 (C-9), 172.2 (C-1), 197.3 (C-2′). HREIMS *m/*z (M+H^+^): Calculated for C_13_H_11_O_4_, 231.0657; found: 231.0672.

Compound **22** was obtained as a brown solid in 69% yield, using a procedure similar to that described for compound **21**. The structure of this compound is supported by the following data: TLC R_f_ = 0.10 (hexane-ethyl acetate 1:1 v/v). mp. 214.0–215.3 °C. IR (ATR, cm^−1^): 3068, 2922, 1770, 1741, 1702, 1266, 1221, 1054. ^1^H-NMR (400 MHz, CDCl_3_ + DMSO-*d_6_***)** *δ* 4.12 (d, 1H, *J* = 2.4 Hz, H-1′), 6.20 (d, 1H, *J* = 2.4 Hz, H-3), 7.84 (m, 8H, H-4, H-5, H-6, H-7, H-3′, H-4′, H-5′, and H-6′).^13^C-NMR. 55,0 (C-1′), 77.3 (C-3), 124,2 (C-4), 123.1 (C-3′), 123.6 (C-6′), 125.3 (C-7), 125.8 (C-6), 129.5 (C-8), 134.4 (C-4′/C-5′), 136.1 (C-5), 142.1 (C-8′), 142.5 (C-9′), 147.4 (C-9), 169.4 (C-1), 194.9 (C-2’), 196.3 (C-7′). HREIMS *m/*z (M+H^+^): Calculated for C_17_H_11_O_4_, 279.0657; found: 279.0665.

### 3.7. Antiproliferative Activity Assays

The human cell lines U937 (lymphoma) and K562 (myeloid leukemia) were purchased from ATCC (Rockville, MD, USA). They were cultured in a RPMI 1640 medium (Sigma Aldrich, St. Louis, MO, USA) supplemented with 10% fetal bovine serum (LGC, Campinas, Brazil), 100 μg/mL streptomycin and 100 U/mL penicillin at 37 °C under 5.0% CO_2_ atmosphere. To evaluate the antiproliferative activity of compounds **10**–**22**, U937 and K562 cells were grown in 96-well plates (TPP, Trasadingen, Switzerland) at a density of 2.0 × 10^4^ cells per well at different compound concentrations (0–100 µM) and etoposide (VP-16) (0–100 µM). No precipitation was observed for any of the compounds at the assayed concentrations. Cell viability was determined by the modified colorimetric 3-(4,5-dimethylthiazol-2-yl)-2,5-diphenyltetrazolium bromide (MTT) (Sigma Aldrich) assay. After 24 and/or 48 h, MTT (0.5 mg/mL) was added to the wells (2 h, 37 °C), followed by the removal of MTT solution and addition of 100 µL/well of dimethyl suphoxide (DMSO, Sigma Aldrich) to solubilize the formazan crystals. Absorbances were measured at 540 nm. Each analysis was performed in quadruplicate and the results were normalized considering the cultures treated with the vehicle alone (1% DMSO). The data were analyzed as the mean ± SD and IC_50_ was determined using GraphPad Prism 5.0 software.

## 4. Conclusions

We have demonstrated that several isobenzofuranone possessing alicyclic and aromatic groups attached to C-3 position exhibit significant antiproliferative active against two cancer cell lines. In some cases, the biological effect was higher than that displayed by the commercial drug etoposide (VP-16). The *in silico* favorable drug-like parameters predicted and shown above suggest that the isobenzofuranones prepared in this work possess good physicochemical properties that qualify them as promising candidates for future studies. Further investigations to validate their potential as anti-tumor compounds, including evaluation of their toxicity against human PMBC cells and in vivo assays using murine animal models, are under way and will be published elsewhere.

## Figures and Tables

*Reagents and Conditions*: (i) DBU, CHCl_3_, reflux; (ii) DBU, CHCl_3_, r.t.; (iii) DBU, CH_3_CN, reflux; (iv) Hg(OAc)_2_, NaOAc, CH_3_COOH, reflux; (v) DMAP, Ac_2_O, CHCl_3_, r.t.

**Table 1:** Inhibitory effects (%) ^a^ of compounds **10**–**22** on K562 cell viability (mean ± SD; n = 4).

Comp.	1 µM	50 µM	100 µM
**10**	44.16 ± 3.59	41.71 ± 1.01	26.71 ± 3.91
**11**	49.69 ± 0.68	45.85 ± 3.07	36.18 ± 1.00
**12**	31.42 ± 1.18	38.38 ± 3.04	38.54 ± 4.24
**13**	32.87 ± 3.20	23.93 ± 3.04	31.64 ± 2.93
**14**	37.71 ± 2.20	49.33 ± 4.70	56.82 ± 1.36
**15**	40.59 ± 2.47	44.32 ± 1.08	43.64 ± 3.85
**16**	20.33 ± 1.13	65.61 ± 2.42	77.73 ± 2.01
**17**	26.77 ± 0.99	56.10 ± 3.58	63.70 ± 0.21
**18**	37.88 ± 3.86	69.63 ± 0.92	80.05 ± 0.28
**19**	12.48 ± 3.57	54.56 ± 2.97	71.76 ± 3.73
**20**	39.87 ± 4.43	43.86 ± 1.92	49.38 ± 2.50
**21**	20.64 ± 1.91	18.60 ± 2.65	21.84 ± 3.39
**22**	46.11 ± 4.1	45.9 ± 3.39	37.61 ± 0.38

^a^ inhibition (%) after 48 h of continuous treatment (n = 4), normalized against K562 cells treated with 1% DMSO (n = 4).

**Table 2:** Inhibitory effects (%) ^a^ of compounds **10**–**22** on U937 cell viability (mean ± SD; n = 4).

Comp.	1 µM	50 µM	100 µM
**10**	28.00 ± 2.75	30.73 ± 2.12	28.81 ± 2.05
**11**	32.34 ± 1.93	28.58 ± 1.62	29.43 ± 2.76
**12**	20.38 ± 3.04	27.01 ± 3.68	24.72 ± 1.48
**13**	26.75 ± 2.12	45.31 ± 1.91	39.10 ± 0.92
**14**	25.57 ± 2.50	31.10 ± 3.53	29.86 ± 1.70
**15**	35.03 ± 2.05	27.03 ± 0.61	22.37 ± 1.13
**16**	11.64 ± 0.28	58.86 ± 2.62	90.82 ± 0.43
**17**	33.87 ± 0.92	61.33 ± 1.98	83.91 ± 0.92
**18**	36.63 ± 1.98	67.68 ± 1.77	90.78 ± 0.21
**19**	23.14 ± 3.19	25.46 ± 3.13	23.12 ± 3.43
**20**	24.33 ± 0.47	30.98 ± 1.04	34.14 ± 0.49
**21**	5.72 ± 1.27	21.17 ± 1.48	29.29 ± 2.02
**22**	27.63 ± 0.92	31.85 ± 0.99	33.58 ± 2.71

^a^ inhibition (%) after 48 h of continuous treatment (n = 4), normalized against U937 cells treated with 1% DMSO (n = 4).

**Table 3:** IC_50_ values (µM) ^a^ obtained when K562 and U937 cells were treated with compounds **16**–**18** and positive control etoposide (VP16).

Cell line	Comp. 16	Compound 17	Compound 18	Etoposide (VP16)
K562	2.79	66.81	1.71	7.06
U937	62.97	71.39	46.63	0.35

^a^ IC_50_: compound concentration required to inhibit 50% of cell viability, determined after 48 h of continuous treatment (n = 4).

**Table 4:** Predicted drug-likeness properties and toxicity risks of compounds **10**–**22** and epotoside calculated by Osiris package.

Comp.	*Bioavailability and Drug Score*							Lipinski’s violations	*Toxicity risks ^a^*			
	cLogP	LogS	MW	HBD	HBA	Drug-likeness	Drug-Score		M	T	I	R
**10**	0.64	−1.68	150.0	1	3	−4.65	0.49	0	-	-	-	-
**11**	1.78	−2.71	244.0	1	4	−6.63	0.46	0	-	-	-	-
**12**	1.98	−2.87	258.0	1	4	−3.61	0.46	0	-	-	-	-
**13**	2.78	−3.30	286.0	1	4	−4.20	0.43	0	-	-	-	-
**14**	2.55	−2.66	242.0	2	4	−4.26	0.46	0	-	-	-	-
**15**	2.87	−3.01	256.0	2	4	−5.25	0.44	0	-	-	-	-
**16**	3.65	−3.53	284.0	2	4	−6.33	0.39	0	-	-	-	-
**17**	3.10	−3.54	326.0	2	6	−6.34	0.40	0	-	-	-	-
**18**	3.41	−3.88	340.0	2	6	−7.52	0.38	0	-	-	-	-
**19**	4.19	−4.41	368.0	2	6	−8.59	0.32	0	-	-	-	-
**20**	2.25	−3.05	272.0	1	4	−3.58	0.45	0	-	-	-	-
**21**	1.29	−2.42	230.0	0	4	−3.95	0.48	0	-	-	-	-
**22**	2.32	−4.04	278.0	0	4	−4.12	0.41	0	-	-	-	-
**Etoposide**	0.53	−3.95	588.0	3	13	−0.28	0.39	2	-	-	-	-

cLogP, calculated lipophilicity; LogS, logarithm of aqueous solubility measured in mol.L^−1^; MW, molecular weight; HBD, number of hydrogen bond donor; HBA, number of hydrogen bond acceptor; M, mutagenic effect; T, tumorigenic effect; I, irritating effect; R, reproductive effect. ^a^ Ranked according to: (−), no bad effect; (±), medium bad effect; (+), bad effect.

**Table 5:** Drug-likeness calculations of compounds **10**–**22** by using Molinspiration package.

Comp.	Molinspiration calculations					Calculations of Bioactivity Scores ^a^					
	cLogP	TPSA ^b^	NONI ^c^	Volume	nRotB	GPCRL	ICM	KI	NRL	PI	EI
**10**	0.895	46.533	1	126.015	0	−0.59	−0.04	−1.12	−0.62	−0.95	0.14
**11**	1.741	63.604	1	211.979	1	−0.60	−0.30	−1.40	−0.42	−0.68	−0.11
**12**	1.982	63.604	1	228.566	1	−0.58	−0.41	−1.35	−0.32	−0.60	−0.14
**13**	3.483	63.604	1	261.955	2	−0.39	−0.29	−1.15	−0.14	−0.35	−0.08
**14**	3.016	66.761	2	205.442	1	−0.29	−0.42	−0.31	−0.20	−0.56	−0.17
**15**	3.416	66.761	2	222.003	1	−0.29	−0.49	−0.30	−0.17	−0.56	−0.21
**16**	4.48	66.761	2	255.392	2	−0.13	−0.37	−0.20	0.03	−0.37	−0.11
**17**	2.183	100.903	2	243.409	3	−0.01	−0.34	−0.18	−0.01	−0.25	−0.08
**18**	2.584	100.903	2	259.970	3	−0.04	−0.41	−0.21	−0.01	−0.27	−0.14
**19**	3.647	100.903	2	293.359	4	0.03	−0.32	−0.19	0.08	−0.17	−0.07
**20**	2.394	63.604	1	244.803	1	−0.50	−0.39	−1.31	−0.27	−0.66	−0.20
**21**	0.652	60.447	0	195.554	1	−0.26	−0.20	−0.85	−0.27	−0.49	0.04
**22**	2.394	60.447	0	233.259	1	−0.12	−0.35	−0.54	−0.05	−0.45	0.09
**^d^ Etop.**	0.698	160.861	3	493.508	5	0.18	−0.48	−0.38	−0.33	0.12	0.30

^a^ GPCRL: GPCR ligand; ICM: Ion channel modulator; KI: Kinase inhibitor; NRL: Nuclear receptor ligand; PI: Protease inhibitor; EI: Enzyme inhibitor; ^b^ TPSA: Total polar surface area; ^c^ NONI: number OH-NH interaction; ^d^ Etop: Etoposide.
